# Supplementary material for: Short- and Long-Term Survival among Elderly Colorectal Cancer Patients in Finland, 2006–2015: A Nationwide Population-Based Registry Study
Source: Cancers (Basel). 2023 Dec 27;16(1):135. doi: 10.3390/cancers16010135 (PMC10777947; doi:10.3390/cancers16010135)
Supplement: Supplementary file 1 [file cancers-16-00135-s001.zip › Figure S1.pdf]

A **<75 years**

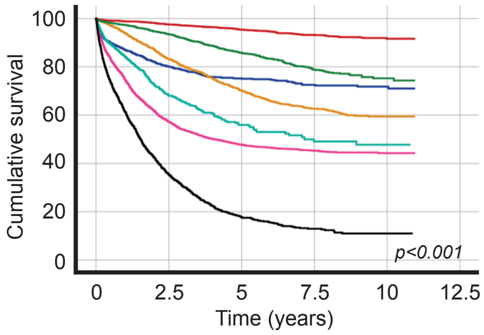

Patients at risk:

FCR 1	2276	2276	1696	967	284
FCR 5	2229	1648	926	429	99
FCR 0	2369	1310	484	245	93
FCR 2	2562	1711	936	426	131
FCR 4	1164	495	129	56	16
FCR 3	2857	1557	1220	661	126
FCR 6	1800	470	132	50	9

B **75–79 years**

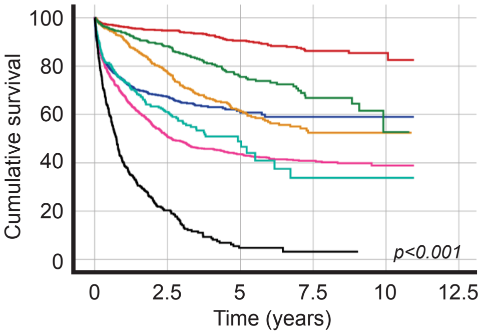

Patients at risk:

FCR 1	776	584	377	73	33
FCR 5	550	341	146	59	5
FCR 0	807	325	92	36	13
FCR 2	557	314	148	54	18
FCR 4	321	126	22	6	1
FCR 3	930	433	321	151	19
FCR 6	346	47	7	2	0

C **80–84 years**

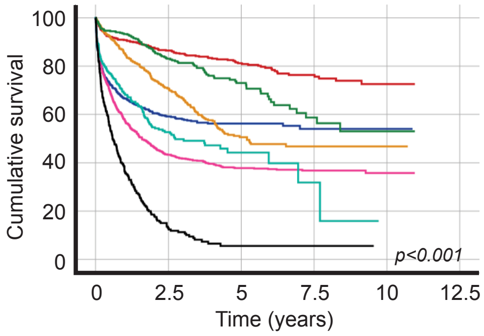

Patients at risk:

FCR 1	643	405	243	106	24
FCR 5	456	251	103	26	2
FCR 0	805	286	84	36	9
FCR 2	449	245	95	34	5
FCR 4	311	97	17	3	0
FCR 3	815	301	212	91	10
FCR 6	264	24	5	3	0

D **85–89 years**

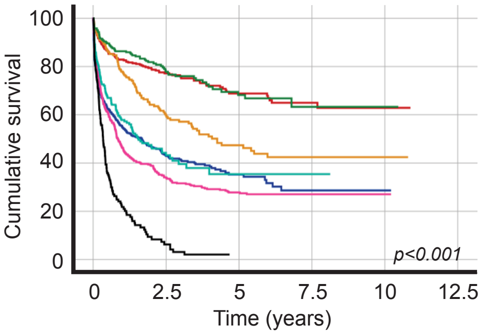

Patients at risk:

FCR 1	382	196	96	37	4
FCR 5	270	131	53	13	1
FCR 0	654	158	39	6	1
FCR 2	237	95	43	15	3
FCR 4	185	43	6	1	0
FCR 3	468	126	77	19	2
FCR 6	146	6	0	0	0

E **≥90 years**

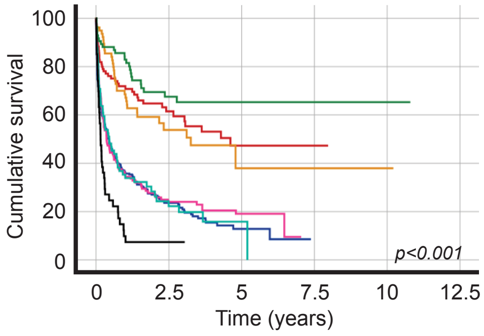

Patients at risk:

FCR 1	117	32	12	1	0
FCR 5	86	33	9	2	1
FCR 0	374	40	5	0	0
FCR 2	80	19	4	1	1
FCR 4	74	11	1	0	0
FCR 3	154	26	9	0	0
FCR 6	47	1	0	0	0

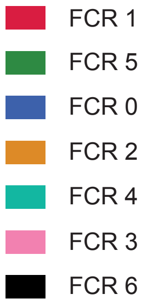

Figure S1. Disease-specific survival analysis for colorectal cancer patients diagnosed in 2006–2015 according to age at diagnosis: (A) <75, (B) 75–79, (C) 80–84, (D) 85–89, and (E) ≥90. Finnish Cancer Registry classes: 0, unknown; 1, localized; 2, non-localized, regional lymph node metastasis only; 3, metastasized further than to regional lymph nodes or invading adjacent tissues; 4, non-localized, no information on extent; 5, locally advanced, tumor invasion to adjacent tissues; and 6, non-localized, including distant lymph node metastasis. The p-value calculated using the log-rank test.
