# Supplementary material for: Short- and Long-Term Survival among Elderly Colorectal Cancer Patients in Finland, 2006–2015: A Nationwide Population-Based Registry Study
Source: Cancers (Basel). 2023 Dec 27;16(1):135. doi: 10.3390/cancers16010135 (PMC10777947; doi:10.3390/cancers16010135)
Supplement: Supplementary file 1 [file cancers-16-00135-s001.zip › Figure S2.pdf]

A **<75 years**

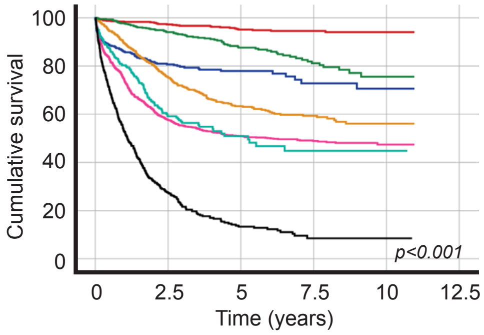

Patients at risk:

FCR 1	660	533	401	229	83
FCR 5	626	444	246	110	30
FCR 0	486	281	97	46	20
FCR 2	716	422	229	118	33
FCR 3	905	500	416	230	48
FCR 4	335	124	40	20	8
FCR 6	495	102	31	8	2

B **75–79 years**

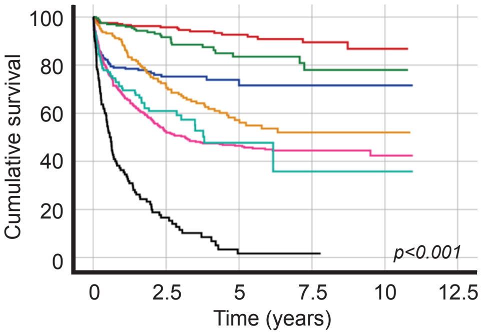

Patients at risk:

FCR 1	246	185	114	55	10
FCR 5	200	125	55	24	2
FCR 0	232	94	31	11	4
FCR 2	208	110	52	20	6
FCR 3	355	169	132	71	10
FCR 4	124	44	7	1	1
FCR 6	111	13	1	1	0

C **80–84 years**

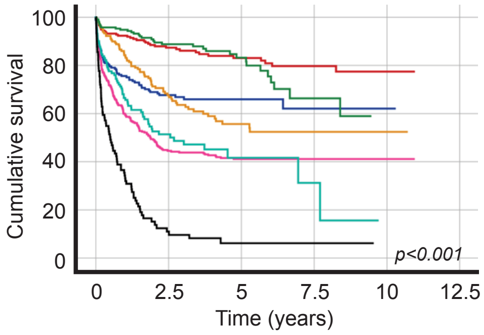

Patients at risk:

FCR 1	245	149	90	41	10
FCR 5	216	121	54	10	0
FCR 0	237	98	28	10	4
FCR 2	183	89	38	11	3
FCR 3	328	125	92	40	7
FCR 4	132	39	7	2	0
FCR 6	91	8	3	2	0

D **85–89 years**

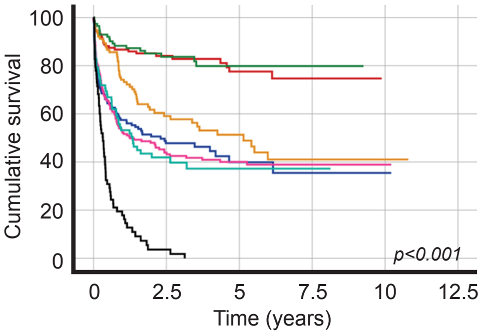

Patients at risk:

FCR 1	151	73	38	15	0
FCR 5	119	59	22	7	0
FCR 0	199	51	14	3	1
FCR 2	116	45	22	9	2
FCR 3	183	64	39	7	2
FCR 4	82	20	4	1	0
FCR 6	64	2	0	0	0

E **≥90 years**

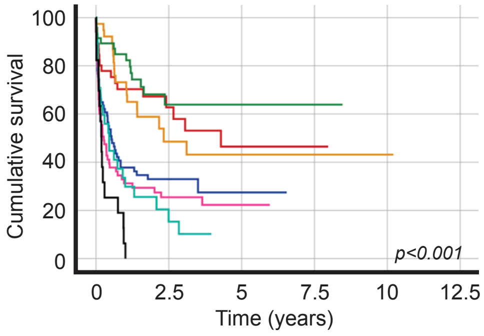

Patients at risk:

FCR 1	48	13	4	1	0
FCR 5	48	14	5	1	0
FCR 0	85	12	1	0	0
FCR 2	39	9	4	1	1
FCR 3	71	13	5	0	0
FCR 4	29	3	0	0	0
FCR 6	17	0	0	0	0

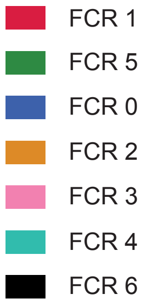

Figure S2. Disease-specific survival analysis for right-sided colon cancer patients diagnosed in 2006–2015 according to age at diagnosis: (A) <75, (B) 75–79, (C) 80–84, (D) 85–89, and (E) ≥90. Finnish Cancer Registry classes: 0, unknown; 1, localized; 2, non-localized, regional lymph node metastasis only; 3, metastasized further than to regional lymph nodes or invading adjacent tissues; 4, non-localized, no information on extent; 5, locally advanced, tumor invasion to adjacent tissues; and 6, non-localized, including distant lymph node metastasis. The p-value calculated using the log-rank test.
