# Supplementary material for: Short- and Long-Term Survival among Elderly Colorectal Cancer Patients in Finland, 2006–2015: A Nationwide Population-Based Registry Study
Source: Cancers (Basel). 2023 Dec 27;16(1):135. doi: 10.3390/cancers16010135 (PMC10777947; doi:10.3390/cancers16010135)
Supplement: Supplementary file 1 [file cancers-16-00135-s001.zip › Figure S3.pdf]

**A** **<75 years**

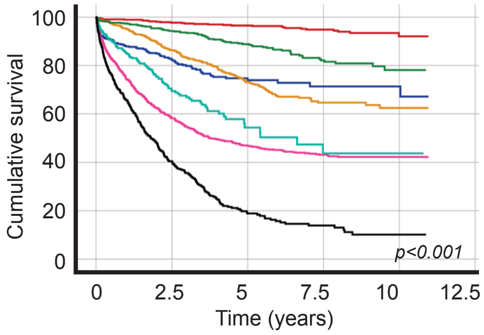


Patients at risk:

| | | | | | |
|---|---|---|---|---|---|
| FCR 1 | 646 | 546 | 426 | 251 | 67 |
| FCR 5 | 655 | 485 | 300 | 150 | 33 |
| FCR 0 | 503 | 274 | 93 | 42 | 17 |
| FCR 2 | 644 | 446 | 253 | 109 | 34 |
| FCR 4 | 299 | 122 | 29 | 12 | 4 |
| FCR 3 | 752 | 419 | 316 | 168 | 28 |
| FCR 6 | 512 | 148 | 41 | 18 | 3 |

**B** **75–79 years**

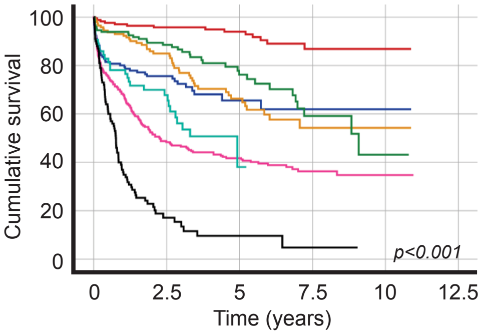


Patients at risk:

| | | | | | |
|---|---|---|---|---|---|
| FCR 1 | 173 | 136 | 92 | 37 | 9 |
| FCR 5 | 149 | 100 | 47 | 19 | 1 |
| FCR 0 | 158 | 79 | 20 | 8 | 5 |
| FCR 2 | 145 | 89 | 45 | 14 | 5 |
| FCR 4 | 64 | 30 | 2 | 0 | 0 |
| FCR 3 | 238 | 106 | 80 | 33 | 6 |
| FCR 6 | 101 | 10 | 3 | 1 | 0 |

**C** **80–84 years**

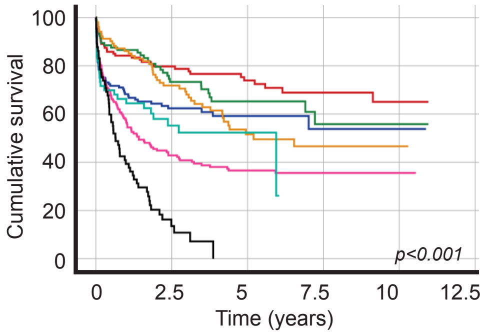


Patients at risk:

| | | | | | |
|---|---|---|---|---|---|
| FCR 1 | 130 | 80 | 52 | 24 | 9 |
| FCR 5 | 105 | 57 | 23 | 11 | 1 |
| FCR 0 | 154 | 57 | 20 | 9 | 2 |
| FCR 2 | 105 | 60 | 26 | 14 | 2 |
| FCR 4 | 63 | 20 | 4 | 0 | 0 |
| FCR 3 | 178 | 64 | 48 | 20 | 1 |
| FCR 6 | 65 | 5 | 0 | 0 | 0 |

**D** **85–89 years**

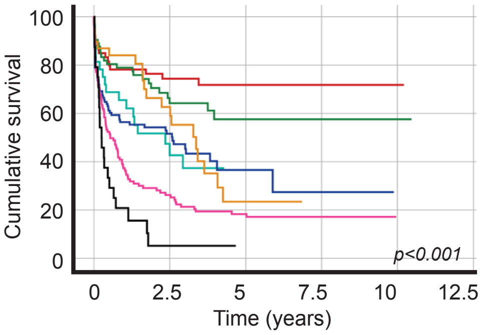


Patients at risk:

| | | | | | |
|---|---|---|---|---|---|
| FCR 1 | 68 | 35 | 21 | 4 | 1 |
| FCR 5 | 75 | 30 | 13 | 4 | 1 |
| FCR 0 | 119 | 37 | 6 | 1 | 0 |
| FCR 2 | 39 | 17 | 3 | 0 | 0 |
| FCR 4 | 33 | 9 | 0 | 0 | 0 |
| FCR 3 | 119 | 27 | 16 | 5 | 0 |
| FCR 6 | 24 | 1 | 0 | 0 | 0 |

**E** **≥90 years**

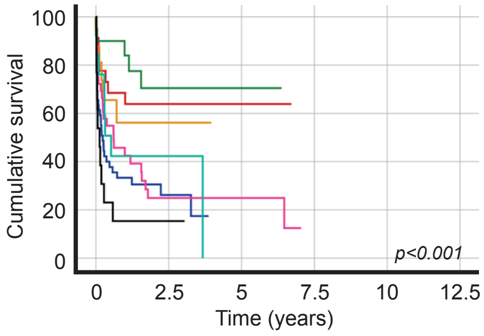


Patients at risk:

| | | | | | |
|---|---|---|---|---|---|
| FCR 1 | 23 | 9 | 6 | 0 | 0 |
| FCR 5 | 20 | 9 | 1 | 0 | 0 |
| FCR 0 | 51 | 3 | 0 | 0 | 0 |
| FCR 2 | 17 | 3 | 0 | 0 | 0 |
| FCR 4 | 13 | 2 | 0 | 0 | 0 |
| FCR 3 | 36 | 6 | 2 | 0 | 0 |
| FCR 6 | 13 | 1 | 0 | 0 | 0 |

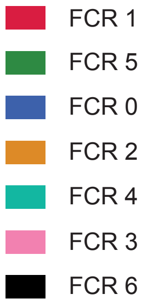

**Figure S3.** Disease-specific survival analysis for left-sided colon cancer patients diagnosed in 2006–2015 according to age at diagnosis: (A) <75, (B) 75–79, (C) 80–84, (D) 85–89, and (E) ≥90. Finnish Cancer Registry classes: 0, unknown; 1, localized; 2, non-localized, regional lymph node metastasis only; 3, metastasized further than to regional lymph nodes or invading adjacent tissues; 4, non-localized, no information on extent; 5, locally advanced, tumor invasion to adjacent tissues; and 6, non-localized, including distant lymph node metastasis. The p-value calculated using the log-rank test.
