# Supplementary material for: Short- and Long-Term Survival among Elderly Colorectal Cancer Patients in Finland, 2006–2015: A Nationwide Population-Based Registry Study
Source: Cancers (Basel). 2023 Dec 27;16(1):135. doi: 10.3390/cancers16010135 (PMC10777947; doi:10.3390/cancers16010135)
Supplement: Supplementary file 1 [file cancers-16-00135-s001.zip › Figure S4.pdf]

A **<75 years**

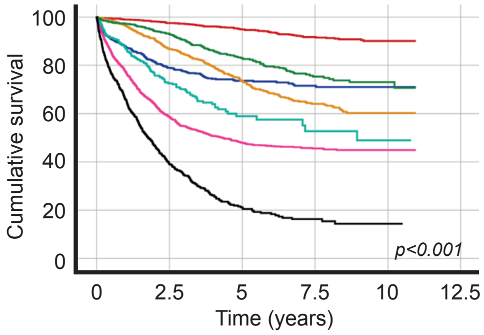

Patients at risk:

FCR 1	1383	1126	819	466	128
FCR 0	1071	574	236	128	42
FCR 5	888	678	368	167	36
FCR 2	1125	795	430	187	61
FCR 4	413	189	47	20	4
FCR 3	1076	595	460	248	46
FCR 6	683	200	58	23	4

B **75–79 years**

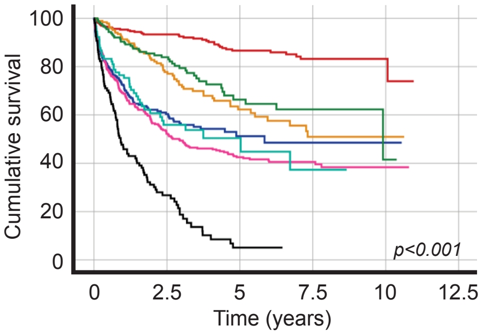

Patients at risk:

FCR 1	327	241	156	77	11
FCR 0	289	99	24	10	3
FCR 5	177	100	38	14	2
FCR 2	192	107	49	19	7
FCR 4	92	33	9	5	0
FCR 3	303	146	101	41	1
FCR 6	110	20	3	0	0

C **80–84 years**

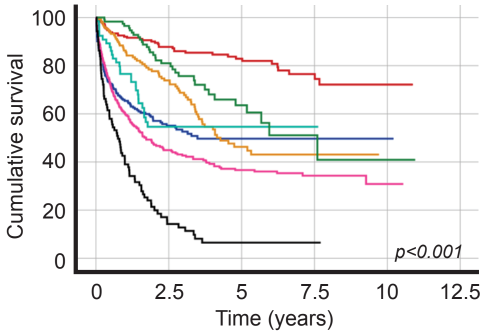

Patients at risk:

FCR 1	237	151	90	38	4
FCR 0	260	77	20	10	1
FCR 5	121	64	25	5	1
FCR 2	150	90	29	9	0
FCR 4	66	19	2	1	0
FCR 3	256	100	66	27	2
FCR 6	88	10	2	1	0

D **85–89 years**

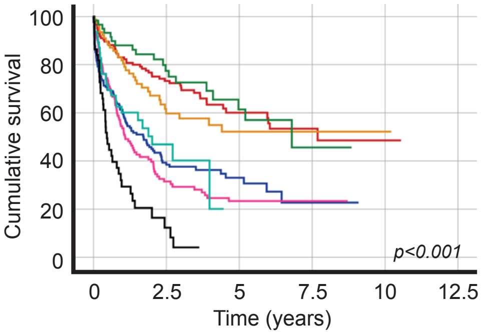

Patients at risk:

FCR 1	148	80	33	15	2
FCR 0	209	45	14	1	0
FCR 5	60	32	15	2	0
FCR 2	78	32	18	6	1
FCR 4	47	11	0	0	0
FCR 3	125	30	19	7	0
FCR 6	44	3	0	0	0

E **≥90 years**

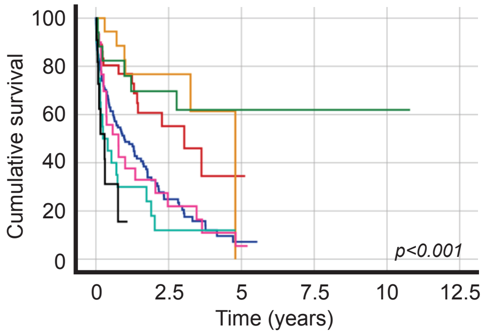

Patients at risk:

FCR 1	37	8	1	0	0
FCR 0	137	17	1	0	0
FCR 5	17	9	3	1	1
FCR 2	18	7	0	0	0
FCR 4	20	2	0	0	0
FCR 3	30	4	1	0	0
FCR 6	11	0	0	0	0

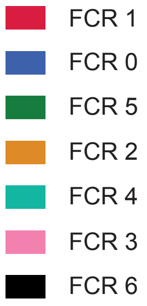

Figure S4. Disease-specific survival analysis for rectal cancer patients diagnosed in 2006–2015 ac-cording to age at diagnosis: (A) <75, (B) 75–79, (C) 80–84, (D) 85–89, and (E) ≥90. Finnish Cancer Registry classes: 0, unknown; 1, localized; 2, non-localized, regional lymph node metastasis only; 3, metastasized further than to regional lymph nodes or invading adjacent tissues; 4, non-localized, no information on extent; 5, locally advanced, tumor invasion to adjacent tissues; and 6, non-localized, including distant lymph node metastasis. The p-value calculated using the log-rank test.
